# Enhancement of THz generation in LiNbO_3_ waveguides via multi-bounce velocity matching

**DOI:** 10.1038/s41377-022-01035-9

**Published:** 2022-11-25

**Authors:** Blake S. Dastrup, Eric R. Sung, Frank Wulf, Clara Saraceno, Keith A. Nelson

**Affiliations:** 1 Department of Chemistry, Massachusetts Institute of Technology, Cambridge, MA 02139 USA; 2 Faculty of Electrical Engineering and Information Technology, Ruhr University Bochum, Bochum, 44801 Germany

**Keywords:** Applied optics, Nonlinear optics

## Abstract

To realize the full promise of terahertz polaritonics (waveguide-based terahertz field generation, interaction, and readout) as a viable spectroscopy platform, much stronger terahertz fields are needed to enable nonlinear and even robust linear terahertz measurements. We use a novel geometric approach in which the optical pump is totally internally reflected to increase the distance over which optical rectification occurs. Velocity matching is achieved by tuning the angle of internal reflection. By doing this, we are able to enhance terahertz spectral amplitude by over 10x compared to conventional single-pass terahertz generation. An analysis of the depletion mechanisms reveals that 3-photon absorption and divergence of the pump beam are the primary limiters of further enhancement. This level of enhancement is promising for enabling routine spectroscopic measurements in an integrated fashion and is made more encouraging by the prospect of further enhancement by using longer pump wavelengths.

## Introduction

Velocity-matched terahertz (THz) generation in LiNbO_3_ (LN) through the use of a spatiotemporally shaped optical tilted pulse front (TPF) has been a key tool for high-field THz generation since it was first used for generation of microjoule-energy THz pulses^1,2^. In this method, the large index mismatch between THz and optical frequencies in LN—which results in noncollinear propagation of the optical pump and THz field generated by the pump—is compensated for by shaping the optical pump to have an angled intensity front^2^. The angle is set to match the Cherenkov angle at which the THz field emanates from the pump^3^, which leads to constructive buildup of the THz pulse. By using TPF pumping, free-space THz E-fields in the MV cm^−1^ range can be reached, which has opened the way for nonlinear THz spectroscopy^4–8^, electron acceleration for coherent x-ray generation^9^, and THz-induced phase transitions^10–13^.

Many studies have been devoted to optimizing generation efficiency, reaching as high as 3.7% optical-to-THz conversion^14–16^, and to understanding the physical mechanisms that limit the efficiency^17,18^ of TPF THz generation. In particular, angular dispersion introduced by the grating used to create the pulse front tilt limits the spatial extent over which THz generation can occur. At the same time, some effort to develop alternative velocity matching schemes has been made, resulting in a handful of notable demonstrations^19–21^.

**Fig. 1:**
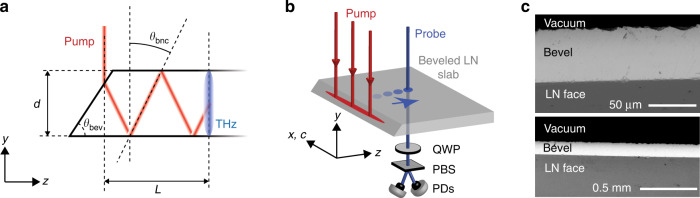
Experimental geometry and sample images. **a** Schematic illustration of beveled LN slab with thickness *d*. Pump light is incident at the bevel angle *θ*_bev_ with respect to the bevel normal and refracts into the crystal at “bounce” angle *θ*_bnc_. Buildup distance for THz generation is denoted as *L*. **b** Diagram of experimental geometry. The LN slab is oriented with the optic axis (c-axis) parallel to the beveled edge. The c-polarized pump pulse is fixed on the bevel and the THz field is sampled at multiple distances from the bevel. After passing through the sample the probe pulse is sent through a quarter wave plate (QWP) and a polarizing beamsplitter (PBS), and then measured on balanced photodiodes (PDs). **c** SEM images of beveled edge of LN slab at two different scales. The slab is viewed from the top down (the *y*-direction as defined in (**b**)). The light gray region shows the beveled edge and the dark gray region below it shows the face of the LN slab

Here we demonstrate a novel velocity matching scheme in LN in which the optical pump beam undergoes repeated total internal reflection (TIR) between the parallel faces of a slab of LN, following a back-and-forth trajectory with an in-plane wavevector that satisfies the velocity matching condition such that the optical beam progresses laterally within the slab at the same speed as the THz wave that it generates. We demonstrate this scheme using thin (50–100 μm) LN slabs with beveled edges through which pump light is coupled into the crystal as illustrated in Fig. 1a. LN slabs in the 10–100 μm thickness range have been exploited as dielectric waveguides for THz fields, enabling the development of a “THz polaritonics” platform (so named because strong coupling between the THz electromagnetic waves and polar optical phonons results in the formation of phonon-polariton modes in LN)^22^ that includes direct visualization of THz fields^23^ and extensive control over THz waveforms through both spatial and temporal shaping of optical pump light^24,25^. This platform has also been used to demonstrate interactions between the THz fields and THz-frequency photonic bandgap structures among other signal processing functionalities^26^ and strong light-matter coupling involving THz magnon modes^27^ in very compact experimental geometries without coupling the THz field out of the waveguide. In the present work, we use the LN waveguide to localize not only the THz field but the optical pump field as well. This allows us to effectively reuse the pump light through its many traversals back and forth through the LN slab, resulting in a THz field buildup similar to that achieved using the separate parts of a tilted pulse front beam to pump successive LN regions. The resulting enhanced field is highly encouraging for the development of a robust chip-based THz spectroscopy and signal processing platform within the scope of THz polaritonics using relatively low optical pump pulse energy.

## Results

### Velocity matching in LN

THz generation by optical rectification (OR) in LN is a second-order nonlinear optical process with the phase-matching relation,

1 $${{{\boldsymbol{k}}}}_{{\Omega }} = {{{\boldsymbol{k}}}}_\omega - {{{\boldsymbol{k}}}}_{\omega - {{\Omega }}}$$

where ***k***_Ω_ is the wavevector at the THz frequency, Ω, and ***k***_ω_ and ${{{\boldsymbol{k}}}}_{\omega - {{\Omega }}}$ are the wavevectors at the optical frequencies, *ω* and $\omega - {{\Omega }}$, respectively.

Considering only the projection along the direction of optical pump propagation allows us to express the Cherenkov angle *θ*_c_ between ***k***_Ω_ and ***k***_ω_ in terms of the THz and optical wave parameters,

2 $${\rm{cos}} \theta_{{{\mathrm{C}}}} = \frac{c}{{{{\Omega }}\;n_{{{{\mathrm{THz}}}}}}}\left( {k_{\upomega} - k_{\omega - {{\Omega }}}} \right)$$

where *n*_THz_ is the THz refractive index and *c* is the vacuum speed of light. We assume that the angle between ***k***_ω_ and ${{{\boldsymbol{k}}}}_{\omega - {{\Omega }}}$ is small since ${{\Omega }} \ll \omega$. Recognizing the RHS as the discrete derivative $\partial k/\partial \omega = 1/v_{{{\mathrm{g}}}}^{{{{\mathrm{op}}}}}$ multiplied by $c/n_{{{{\mathrm{THz}}}}} = v_{{{\mathrm{p}}}}^{{{{\mathrm{THz}}}}}$, we arrive at the expression,

3 $$\cos \theta _{{{\mathrm{C}}}} = \frac{{v_{{{\mathrm{p}}}}^{{{{\mathrm{THz}}}}}}}{{v_{{{\mathrm{g}}}}^{{{{\mathrm{op}}}}}}}$$

where $v_{{{\mathrm{g}}}}^{{{{\mathrm{op}}}}}$ is the optical group velocity and $v_{{{\mathrm{p}}}}^{{{{\mathrm{THz}}}}}$ is the THz phase velocity.

In the case of an optical pump beam focused to a round spot, the THz field emanates from the pump as a cone of light at the Cherenkov angle *θ*_c_, so called in reference to Cherenkov radiation, which is isomorphic to the THz generation process described here^3^. In our scheme, the in-plane projection of the pump velocity is given by,

4 $$v_{{\mathrm{g}},z}^{\mathrm{op}} = v_{\mathrm{g}}^{\mathrm{op}}\sin \theta_{\mathrm{bnc}}$$

where $v_{{{{\mathrm{g}}}},z}^{{{{\mathrm{op}}}}}$ is the component of the optical group velocity along the *z*-direction and $\theta _{{{{\mathrm{bnc}}}}}$ is the bounce angle defined in Fig. 1a. When $\theta _{{{{\mathrm{bnc}}}}} = \left( {\pi /2 - \theta _{{{\mathrm{C}}}}} \right),$ substitution of (4) into (3) gives $v_{{{{\mathrm{g}}}},z}^{{{{\mathrm{op}}}}} = v_{{{\mathrm{p}}}}^{{{{\mathrm{THz}}}}}$. In other words, the THz phase velocity is matched to the group velocity of the optical pump in the positive *z*-direction. Waveguide dispersion effectively introduces frequency dependence into the THz phase velocity, $v_{{{\mathrm{p}}}}^{{{{\mathrm{THz}}}}}({{\Omega }})$ (beyond the weak frequency dependence due to material dispersion in LN). In this case, the velocity matching condition becomes,

5 $$\sin [ \theta_{{{{\mathrm{bnc}}}}}({{\Omega }})] = \frac{{v_{{{\mathrm{p}}}}^{{{{\mathrm{THz}}}}}\left( {{\Omega }}\right)}}{{v_{{{\mathrm{g}}}}^{{{{\mathrm{op}}}}}}}$$

which indicates that for a given bounce angle, the velocity matching condition will be satisfied optimally for a particular THz frequency.

Backward-propagating THz signal (i.e. propagating in the negative *z*-direction) is also generated and, while not contributing to the buildup, can be observed in the experiment and leads to an undesirable interference effect that is described below.

### THz polarization and dielectric waveguide dispersion

Because the thickness of the LN slabs used in our experiments is comparable to the wavelengths of THz light confined in the slabs, the THz field propagates in dielectric waveguide modes which are the electromagnetic eigenmodes for a high-index core (LN) sandwiched between layers of a low-index cladding (air). Both transverse-electric (TE) and transverse-magnetic (TM) modes are supported, the former with a continuous-valued electric field across the core/cladding interface and the electric field polarized in the slab plane (see Fig. S1), and the latter with a continuous-valued displacement field across the interface and the magnetic field polarized in the slab plane. Since OR is mediated by the *d*_33_ element of the second-order nonlinear tensor of LN, the optical pump and the emerging THz field are both polarized along the optic axis (c-axis) of LN, which gives rise to exclusive generation of TE modes for our geometry.

**Fig. 2:**
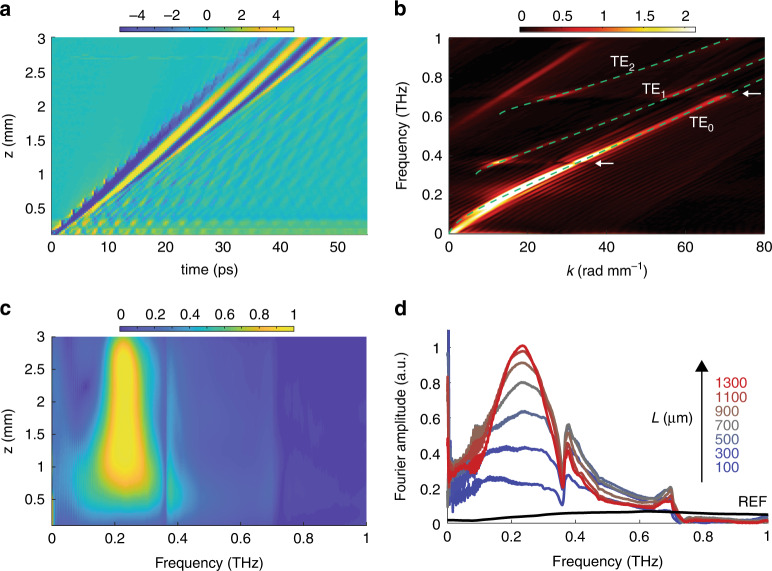
THz data and buildup plots for sample III. **a** Space-time plot of raw data. **b** Dispersion plot obtained by 2D Fourier transform of data in (**a**). Calculated dispersion curves for TE_0_, TE_1_ and TE_2_ dielectric waveguide modes are shown by the dashed curves. White arrows indicate spectral modulations caused by backward-propagating THz waves. **c** Surface plot of THz buildup. **d** 2D plot of THz buildup with selected probe positions shown. Reference shown in black

Dielectric waveguide modes are characterized by dispersion that transitions smoothly from cladding-like to core-like as the wavelength decreases (as depicted in Fig. 2b). As it propagates through the slab, the broadband THz pulse is dispersed, with low frequencies leading high frequencies. Over the course of their interaction, waveguide dispersion creates a velocity mismatch between the optical pump and THz frequency components that either lag behind or lead it. The result is a narrowing of the THz bandwidth over the course of the buildup. By changing *θ*_bnc_, the buildup center frequency can be tuned. For our experiments, *θ*_bnc_ was chosen to match a selected frequency in the TE_0_ mode since this is where the majority of the THz energy resides.

### THz buildup

We measured the THz buildup in five samples with different combinations of thickness and bevel angle chosen to match a particular THz frequency. In each case, the pump bounce angle was matched to a frequency of the TE_0_ mode. Figure 2a shows a representative raw space-time plot, with the corresponding dispersion plot shown in Fig. 2b. The buildup, shown in Fig. 2c, d, was extracted by inverse Fourier transformation of the *k*-dimension after isolation of the TE_0_ waveguide mode. A detailed description of this process is given in Supplementary information. Sample parameters and experimental results are summarized in Table 1.

**Table 1:** Enhancement values and buildup center frequencies

Sample	*d* (μm)	*θ*_bev_ (deg)	$f_0^{{{{\mathrm{theo}}}}}$ (THz)	$f_0^{{{{\mathrm{meas}}}}}$ (THz)	*L*_max_ (mm)	$\bar \eta$	$\eta _0$
I	50	59	0.52	0.50	1.4	35.9	10.8
II	50	53	0.78	0.61	0.9	98.4	13.7
III	100	59	0.26	0.23	1.0	79.2	29.2
IV	100	53	0.40	0.38	0.5	69.9	12.8
V	100	50	0.57	0.46	0.6	113.8	13.4

**Fig. 3:**
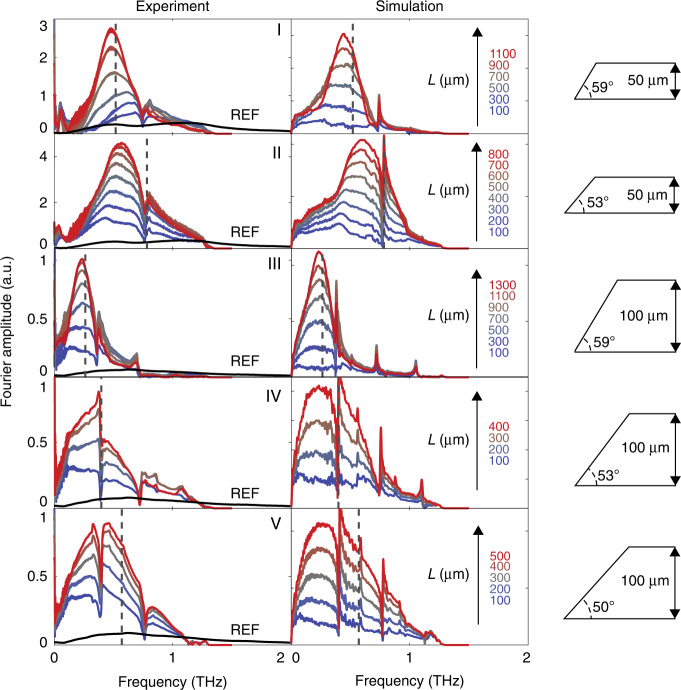
Buildup of THz spectral amplitude from both experiment and simulation at room temperature. The corresponding sample for each row of plots is shown on the right. The theoretical buildup frequency is marked by the vertical grey dotted line in each plot. The legend for each plot gives the selected buildup distances, *L*, from *L* = 100 μm to *L* ≈ *L*_max_

Room-temperature buildup plots for each of the five samples are shown in Fig. 3. Buildup surfaces and dispersion plots are shown for samples I, II, IV, and V in Fig. S4. For each sample we observe a monotonic increase in the THz spectral amplitude over an average buildup distance of ≈1.1 mm, with an average amplitude enhancement of 11x relative to the reference trace at the buildup center frequency (see Table 1).

Experiments were also conducted at low temperature (80 K) to reduce the amount of linear THz absorption. The results were similar to those at room temperature, with some differences including longer average buildup distances due to lower phonon damping rates at 80 K. The details are presented in Supplementary information.

**Fig. 4:**
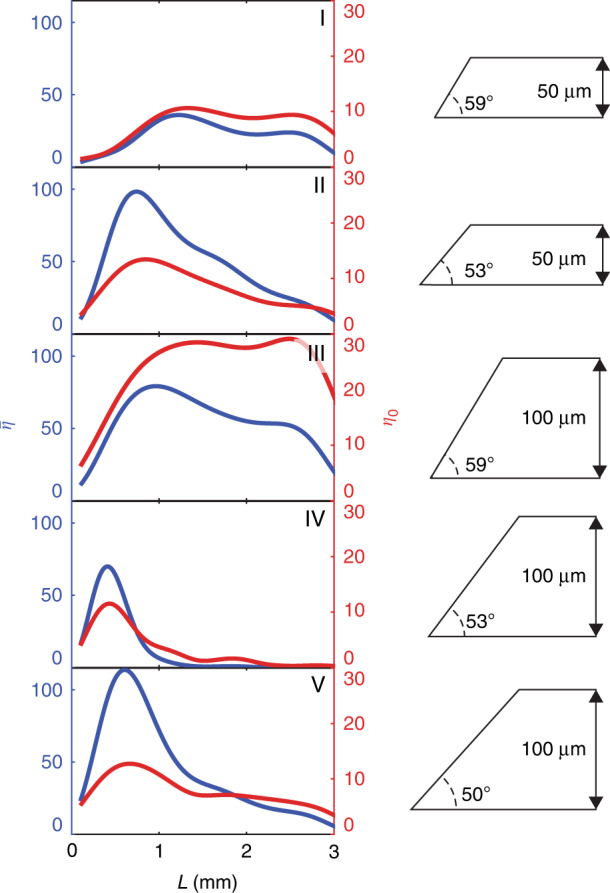
THz buildup plots. Integrated enhancement ($\bar \eta$) in blue, and buildup enhancement ($\eta _{0}$) in red, plotted for each sample as a function of buildup distance (*L*). Sample thickness and angle are shown by the diagram at the right of each plot

To quantify the extent of enhancement obtained, we compared the buildup traces to a reference that was collected by line-focusing the pump onto a portion of the LN slab that was not beveled and measuring the THz signal generated by a single pass of the pump through the slab. The reference was measured in this way to match previous THz polaritonics experiments^22,27^. A scaling factor was applied to the reference spectrum to correct for differences in Fresnel reflections and pump fluence caused by the difference in incidence angle for sample and reference measurements (see Supplementary information for details). The reference spectrum is shown in Fig. 3 as the solid black curve in each of the experimental plots. In calculating the enhancement, we consider both the integrated enhancement,

6 $$\bar \eta = \frac{{\mathop {\int }\nolimits_0^\infty \left| {E\left( \omega \right)} \right|^2n_{{{{\mathrm{THz}}}}}\left( \omega \right)d\omega }}{{\mathop {\int }\nolimits_0^\infty \left| {E_{{{{\mathrm{ref}}}}}\left( \omega \right)} \right|^2n_{{{{\mathrm{THz}}}}}\left( \omega \right)d\omega }}$$

as well as the maximum amplitude enhancement,

7 $$\eta _0 = \frac{{E\left( {\omega _0^{{{{\mathrm{meas}}}}}} \right)}}{{E_{{{{\mathrm{ref}}}}}\left( {\omega _0^{{{{\mathrm{meas}}}}}} \right)}}$$

where $\omega _0^{{{{\mathrm{meas}}}}} = 2\pi f_0^{{{{\mathrm{meas}}}}}$ is the experimental buildup center frequency. The former measures the enhancement in total THz energy over all frequencies and is approximately proportional to the overall THz generation efficiency (see Fig. S6), while the latter measures the enhancement in amplitude at the buildup center frequency. Maximum values of $\bar \eta$ and $\eta _0$ are reported for each sample in Table 1, and plotted as a function of buildup distance, *L*, in Fig. 4. Peak buildup distance, *L*_max_, as given in Table 1 is the distance at which $\eta _0$ is maximized.

## Discussion

### THz buildup trends

For each sample configuration, we see significant buildup of spectral amplitude in the TE_0_ waveguide mode of the LN slab, which we attribute to a prolonged interaction with pump beam facilitated by the TIR geometry. For samples I and II, the pump bounces 48 times (24 back-and-forth traversals) on average before reaching the peak buildup, while for samples III-V, the peak buildup occurs after 29 bounces of the pump on average. The buildup center frequency is determined by the bevel angle and the sample thickness and generally agrees with the value expected from velocity matching considerations. However, we see a slight deviation for samples II and V from the predicted buildup center frequency, $f_0^{{{{\mathrm{theo}}}}}$, in both simulation and experiment. We attribute this difference to interference with backward-propagating THz waves, which gives rise to dips in the buildup spectra that overlap with $f_0^{{{{\mathrm{theo}}}}}$, thereby shifting the observed buildup frequency. A further discrepancy could also arise due to the increasing THz absorption with increasing frequency in LN. In this case, the buildup process is balanced against the frequency-dependent THz absorption. This is borne out by the observation that disagreement between $f_0^{{{{\mathrm{meas}}}}}$ and $f_0^{{{{\mathrm{theo}}}}}$ generally increases with increasing $f_0^{{{{\mathrm{theo}}}}}$, and by the fact that the simulated buildups show disagreement that qualitatively agrees with the experiment.

Sharp spectral modulations that appear as a dip in the THz spectrum can be seen at ~0.75 THz in samples I and II and at ~0.37 THz and ~0.75 THz in samples III-V. The modulations are present even after isolating the TE_0_ mode as described earlier. In the FDTD simulation, it can be seen that backward-propagating THz waves—THz waves generated by the optical pump but traveling initially in the opposite direction of the optical pump—form a zig-zag pattern that reflects from the bevel, creating a train of reflected pulses that follow the main pulse (see Fig. S5). The periodicity of this pulse train gives rise to the sharp modulations in the spectrum that can be seen in Fig. 3, and which are indicated by the white arrows in Fig. 2b. The pulse train gives rise to a relatively flat (constant in frequency) dispersion feature that crosses the TE_0_ mode dispersion at the frequencies indicated above. This dispersion feature results from the fact that the periodicity of the pulse train is not a function of the actual THz frequency content of the THz waves forming the pulse train, but rather of the geometry of the optical pump trajectory. In addition, the dips observed do not represent the true absence of these frequencies from the main THz pulse, but rather the destructive interference of that portion of the main THz spectrum with the reflected THz waves. To illustrate this, buildup plots that were time-windowed to retain only the main THz pulse are shown in Fig. S7.

Further narrowing of the THz spectrum continues even after the buildup peaks. This can be seen clearly in Fig. 2c and Fig. S4. The narrowing indicates that although the pump continues to generate new cycles of the THz field, the conversion efficiency has diminished to the point that THz absorption reduces the overall THz energy at a higher rate than new THz signal is generated.

### THz buildup saturation

**Fig. 5:**
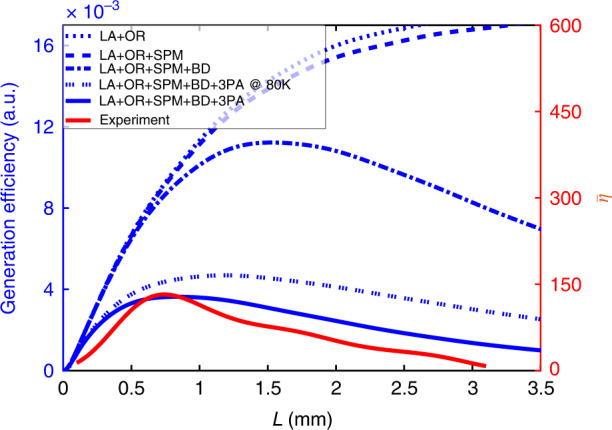
Comparison of generation efficiency for different combinations of depletion mechanisms. The following mechanisms were considered: linear absorption (LA), optical rectification (OR), self-phase modulation (SPM), pump beam divergence (BD), and 3-photon absorption (3PA). Simulated curves are plotted against the left axis, while the experimental integrated enhancement curve is plotted against the right axis. Data shown here are for sample II (*d* = 50 μm, $\theta _{{{{\mathrm{bev}}}}} = 53^\circ$)

The saturation of the THz buildup occurs as a result of pump depletion, dispersion, and THz absorption^17^. In order to understand the roles of these processes in our experimental results, we numerically solved the coupled THz and optical wave equations in one spatial dimension (the direction of THz propagation), which accounts for the different depletion mechanisms at play (optical rectification, 2,3-photon absorption, etc.)^17,18^. The integrated enhancement ($\bar \eta$) is plotted as a function of buildup distance (*L*) in Fig. 5, along with calculated generation efficiency curves with progressing levels of included depletion mechanisms. As noted previously, the calculated efficiency is approximately proportional to $\bar \eta$. As can be seen from the plot, the processes that limit THz generation most strongly are pump beam divergence and 3-photon absorption. The Rayleigh range for the pump beam, assuming a beam waist of 15 μm, is ~0.9 mm. Initially, divergence of the pump beam results in a drop in the pump intensity, which reduces the OR efficiency. After many reflections of the pump, some wavevector components of the diverging beam deviate significantly from the bounce trajectory, which leads to further degeneration of the nonlinear process. In contrast, the limiting mechanism for TPF generation is the combination of grating-induced angular dispersion and OR-induced repeated red-shifting of the pump spectrum^18^. One reason that pump red-shifting plays a less significant role in our case is THz spectral narrowing due to waveguide dispersion described earlier. Narrowing of the THz spectrum leads to a field with multiple cycles that is spatially more delocalized than a single-cycle field, and therefore that interacts with the pump less strongly. The fact that pump beam divergence is a primary limiting factor in saturation of THz buildup suggests that improvements could be made by focusing the beam more loosely (but keeping pump fluence constant). This, in turn, is limited by the bevel aperture. The effective aperture width could be enlarged by directing the pump light into the bevel at normal incidence, though this is more difficult to execute in practice than using refraction as in the present work. Ultimately the pump beam width would be limited by wavevector considerations, i.e. the wavevector content should include the desired wavevector range in the dispersion curves shown in Fig. 2d. Further improvements could be made by using a longer pump wavelength such as the output of an Yb-based femtosecond system to reduce 3-photon absorption^28^.

In summary, we have demonstrated 11-fold enhancement of peak THz spectral amplitude in LN waveguides by using a velocity-matched THz generation scheme based on total internal reflection of the optical pump. The center frequency of the THz buildup can be tuned by changing the bounce angle of the pump. Simulations of experimental results indicate that the primary mechanisms that lead to THz buildup saturation are divergence of the pump beam, which may be improved by directing the pump light into the bevel at normal incidence to make full use of the bevel aperture, and three-photon absorption, which could be eliminated by use of a longer pump wavelength.

These results represent a relatively simple method for accessing greatly enhanced THz spectral amplitudes for THz polaritonics applications. This could prove useful for linear and nonlinear THz signal processing and spectroscopic measurements. Further enhancement may be possible through the use of optical pump fields that are tailored for difference-frequency mixing at a selected THz frequency^29^.

## Materials and methods

### Preparation of beveled LN samples

We used *x*-cut 5% MgO-LN slabs with 50 μm and 100 μm thicknesses (NanoLN). To create the beveled edge, we mounted the slabs on the angled face of a negative epoxy mold (c-axis parallel to polishing plane) and then backfilled the mold with prototyping wax (McMaster-Carr). The LN slabs were then polished with silicon-carbide grinding paper with a minimum grit size of 9 μm at which point the beveled surface of the crystal appeared optically smooth under an optical microscope. SEM images of the bevel for a representative sample are shown in Fig. 1c. For the THz measurements, the slabs were fixed at the edges to a copper mount with a rectangular aperture such that the slabs were effectively free standing.

### Experimental setup

Optical measurements were made using the 1 kHz repetition rate output of a Ti:Sapphire regenerative amplifier providing 1.5 mJ pulses centered at 800 nm with 100 fs duration. The output was split 95:5 into pump and probe respectively. The pump was attenuated to an overall pulse energy of 200 μJ and an optical chopper running at 500 Hz was used to modulate the pump for electro-optic (EO) detection. The pump was polarized parallel to the LN c-axis and cylindrically focused onto the bevel with a 20 cm cylindrical lens. The pump was incident normal to the slab face (oblique to the beveled face) and refracted into the crystal at angle *θ*_bnc_. The THz field was measured by EO sampling directly in the LN slab. This method of detection is typical for LN waveguide THz measurements, and has been described elsewhere^27^. See Fig. 1b. The probe beam was frequency-doubled in *β*-barium borate (BBO) and then polarized 45° relative to the optic axis of LN before being focused onto the back face of the sample with a 15 cm lens. After passing through the sample, the probe was separated from the pump beam path with a dichroic mirror, then directed through a λ/4 plate and a Wollaston prism and detected on two balanced photodiodes. A delay stage in the pump line was used to scan the pump-probe delay. To measure the THz buildup, the probe position was shifted in the *z*-direction in 25 μm increments from a starting distance of 100 μm to a maximum distance of 3 mm away from the bevel.

In order to better visualize the buildup in the TE_0_ mode, we isolated the portion of the dispersion curve that corresponds to the TE_0_ mode using an envelope function (see Supplementary information). The spatial buildup plot shown in Fig. 2c was then obtained by inverse Fourier transformation over the spatial dimension of the isolated TE_0_ dispersion. Figure 2d shows THz spectra at selected probe locations from the data in Fig. 2c.

### Simulations

We performed simulations of the experiments using the finite-difference time-domain (FDTD) method. The optical pump was treated as a moving point source that followed the back-and-forth trajectory shown in Fig. 1a. The THz field emitted by each point source at each time step was calculated using a one-dimensional numerical simulation of the coupled optical/THz wave equations, following the method of Ravi et al.^18^. For more information about simulation methods, see the Supplementary information.

## Supplementary information

Supplementary Information

## References

[1] Yeh KL (2007). Generation of 10μJ ultrashort terahertz pulses by optical rectification. Appl. Phys. Lett..

[2] Hebling J (2002). Velocity matching by pulse front tilting for large-area THz-pulse generation. Opt. Express.

[3] Auston DH (1984). Cherenkov radiation from femtosecond optical pulses in electro-optic media. Phys. Rev. Lett..

[4] Hebling J (2008). High-power THz generation, THz nonlinear optics, and THz nonlinear spectroscopy. IEEE J. Sel. Top. Quantum Electron..

[5] Hwang HY (2015). A review of non-linear terahertz spectroscopy with ultrashort tabletop-laser pulses. J. Mod. Opt..

[6] Lu J (2018). Two-dimensional spectroscopy at terahertz frequencies. Top. Curr. Chem..

[7] Baierl S (2016). Nonlinear spin control by terahertz-driven anisotropy fields. Nat. Photonics.

[8] Elsaesser, T., Reimann, K. & Woerner, M. *Concepts and Applications of Nonlinear Terahertz Spectroscopy* (San Rafael: Morgan & Claypool Publishers, 2019).

[9] Kärtner FX (2020). Terahertz accelerator based electron and x-ray sources. TST.

[10] Liu MK (2012). Terahertz-field-induced insulator-to-metal transition in vanadium dioxide metamaterial. Nature.

[11] Li X (2019). Terahertz field–induced ferroelectricity in quantum paraelectric SrTiO_3_. Science.

[12] Schlauderer S (2019). Temporal and spectral fingerprints of ultrafast all-coherent spin switching. Nature.

[13] Shi, J. J. et al. Terahertz-driven irreversible topological phase transition in two-dimensional MoTe_2_. Preprint at https://arxiv.org/abs/1910.13609v1 (2019).

[14] Huang SW (2013). High conversion efficiency, high energy terahertz pulses by optical rectification in cryogenically cooled lithium niobate. Opt. Lett..

[15] Huang WR (2015). Highly efficient terahertz pulse generation by optical rectification in stoichiometric and cryo-cooled congruent lithium niobate. J. Mod. Opt..

[16] Fülöp JA (2014). Efficient generation of THz pulses with 0.4 mJ energy. Opt. Express.

[17] Ravi K (2015). Theory of terahertz generation by optical rectification using tilted-pulse-fronts. Opt. Express.

[18] Ravi K (2014). Limitations to THz generation by optical rectification using tilted pulse fronts. Opt. Express.

[19] Ofori-Okai BK (2016). THz generation using a reflective stair-step echelon. Opt. Express.

[20] Pálfalvi L (2008). Novel setups for extremely high power single-cycle terahertz pulse generation by optical rectification. Appl. Phys. Lett..

[21] Tsubouchi M (2014). Contact grating device with Fabry–Perot resonator for effective terahertz light generation. Opt. Lett..

[22] Feurer T (2007). Terahertz polaritonics. Annu. Rev. Mater. Res..

[23] Wu Q (2009). Quantitative phase contrast imaging of THz electric fields in a dielectric waveguide. Opt. Express.

[24] Feurer T, Vaughan JC, Nelson KA (2003). Spatiotemporal coherent control of lattice vibrational waves. Science.

[25] Feurer T (2004). Typesetting of terahertz waveforms. Opt. Lett..

[26] Ofori-Okai BK (2014). Direct experimental visualization of waves and band structure in 2D photonic crystal slabs. N. J. Phys..

[27] Sivarajah P (2019). THz-frequency magnon-phonon-polaritons in the collective strong-coupling regime. J. Appl. Phys..

[28] Hoffmann MC (2007). Efficient terahertz generation by optical rectification at 1035 nm. Opt. Express.

[29] Chen Z (2011). Generation of high power tunable multicycle teraherz pulses. Appl. Phys. Lett..

