# Supplementary material for: Enhancement of THz generation in LiNbO3 waveguides via multi-bounce velocity matching
Source: Light Sci Appl. 2022 Nov 25;11:335. doi: 10.1038/s41377-022-01035-9 (PMC9700704; doi:10.1038/s41377-022-01035-9)
Supplement: Supplementary file 1 — Supplementary Information [file 41377_2022_1035_MOESM1_ESM.docx]

**Supplementary Information for Enhancement of THz Generation in LiNbO_3_ Waveguides via Multi-Bounce Velocity Matching**

Blake S. Dastrup^1^*, Eric R. Sung^1^*, Frank Wulf^2^, Clara Saraceno^2^, Keith A. Nelson^1‡^

*^1^Department of Chemistry, Massachusetts Institute of Technology, Cambridge, MA, USA 02139*

*^2^Faculty of Electrical Engineering and Information Technology, Ruhr University Bochum, Bochum, Germany 44801*

**Contents**

1. **Dielectric Waveguide Modes**
2. **Reference Scaling and Isolation of TE_0_ Waveguide Mode**
3. **Low Temperature Experiments**
4. **Room Temperature Buildup Surfaces and Dispersion Plots**
5. **Simulation**
6. **Geometric Explanation of Modulation Frequencies**
7. **Estimation of Overall THz Generation Efficiency**
8. **Time-Windowed Buildup Plots**

**References**

**S.1 *Dielectric Waveguide Modes***


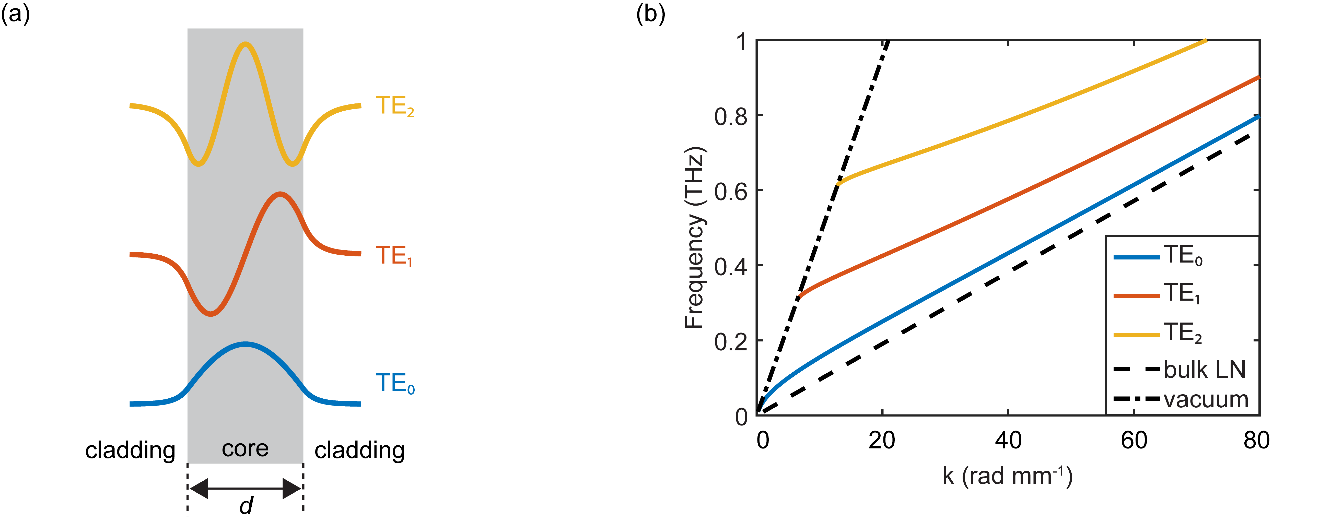
Mode profiles for TE and TM modes of a symmetric dielectric waveguide can be determined analytically. For a full derivation see Reference 1. In our experiments, we see propagation of THz fields in the three lowest order TE waveguide modes of the LN slab (TE­_0_, TE_1_, and TE_2_). Fig. S1 shows the analytical mode profiles and dispersion curves for these modes. Mode profiles are shown for $f=1.0$ THz.

**Figure S1.** (a) E-field profiles at $f=1$ THz and (b) dispersion curves for TE_0_, TE_1_, and TE_2_ dielectric waveguide modes in LN ($n\approx5.1$) with $d=100$ μm. Dispersion curves and mode profiles for $d=50$ μm look similar.

**S.2 *Reference Scaling and Isolation of TE_0_ waveguide mode***

In our measurements, the reference was collected with the pump at normal incidence to the face of the sample, while the sample measurements were collected with the pump at an angle of incidence on the bevel equal to $\theta_{bev}$. To correct for the resulting differences in Fresnel reflection and pump fluence, we scaled the reference THz spectrum as follows,

$\tilde{E}_{\mathrm{ref}}\left( \omega\right)=\alpha_{\mathrm{Frensel}}\alpha_{\mathrm{fluence}}E_{\mathrm{ref}}(\omega)$

where $\tilde{E}_{\mathrm{ref}}(\omega)$ is the corrected reference spectrum, $E_{\mathrm{ref}}\left( \omega\right)$ is the raw reference spectrum, and $\alpha_{\mathrm{Fresnel}}$ {$\alpha_{\mathrm{fluence}}$} is the scaling factor for Fresnel reflection {pump fluence} considerations. These can then be expressed as,

$$\alpha_{\mathrm{Fresnel}}=(1-R_{\theta_{\mathrm{bev}}})/(1-R_{0})$$

$\alpha_{\mathrm{fluence}}=\frac{A_{0}}{A_{\theta_{\mathrm{bev}}}}=\frac{\cos\left( \theta_{\mathrm{bev}} \right)}{\sqrt{1-\frac{1}{n_{\mathrm{LN}}^{2}}\sin^{2} \left( \theta_{\mathrm{bev}} \right)}}$

where $R_{\theta_{\mathrm{bev}}} \{R_{0}\}$ is the power reflection coefficient and $A_{\theta_{\mathrm{bev}}}$ {$A_{0}$} is the pump spot size for $\theta_{\mathrm{inc}}=\theta_{\mathrm{bev}} \{0^{\circ}$}. $n_{\mathrm{LN}}$ is the extraordinary refractive index of LN at 800 nm.

Isolation of the TE_0_ mode for analysis of the buildup was done by applying a window function to the dispersion plot shown in Fig. 2b of the main text. The window function, $w_{k}$, was constructed by fitting the TE_0_ peak to a Lorentzian function for each constant wavenumber slice, $k$, of the dispersion curve. The width and center of the Lorentzian fit were then used as input parameters for a super-Gaussian function of the form,

$$w_{k}\left( f \right)=\exp\left( -\left( \frac{f-f_{0}}{2.25* \Gamma_{\mathrm{FWHM}}} \right)^{20} \right)$$

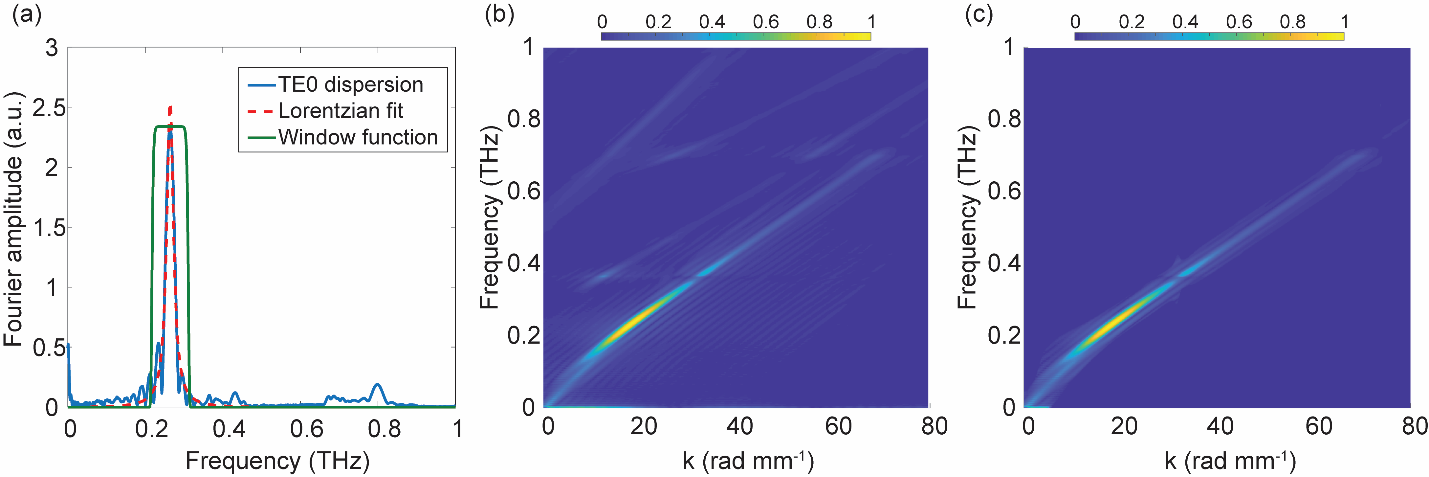
where $f_{0}$ is the center frequency of the Lorentzian fit, and $\Gamma_{\mathrm{FWHM}}$ is the full width at half maximum of the Lorentzian fit. A representative wavenumber slice showing the Lorentzian fit and the super-Gaussian window function is shown in Fig. S2. The windowed dispersion was then obtained by multiplying the raw THz spectrum at each constant wavenumber slice by the window function, $w_{k}(f)$.

**Figure S2.** (a) Constant wavenumber slice of THz dispersion at *k* = 21.5 rad/m^­^m (blue). Lorentzian fit is indicated by the red dashed line, and the super-Gaussian window function is shown in green. Note: for viewing convenience the window function has been scaled to the TE_0_ peak maximum, but the actual value of the window function maximum is 1. (b) Dispersion plot obtained by 2D Fourier transformation of raw space-time data. (c) Cropped TE_0_ dispersion obtained by multiplying the dispersion plot in (b) by the window function obtained from the Lorentzian fitting procedure shown in (a) for every wavenumber.

**S.3 *Low Temperature Experiments***

We performed experiments at 80 K to investigate the THz buildup where THz absorption in LN is known to be lower than at room temperature^2^. In these experiments, the LN slab was fixed to a copper mount using silver paste to ensure good thermal contact. The copper mount was mounted sample cryostat and cooled with liquid nitrogen. Values for $\bar{\eta}$ and $\eta_{0}$ are listed in Table S1, and are generally not higher at 80K than at room temperature. On the other hand, at 80K the THz buildup peaks at longer distances ($L$) than at room temperature for all samples except sample I. For samples IV and V the buildup peaks at a substantially longer distance (see Fig. S3). The absence of an increase in overall THz spectral energy is unexpected given the sizeable improvements in THz yield observed for TPF generation at low temperature^3^, however, simulation predicts only a modest improvement for the multi-bounce geometry (see Fig. 5 in the main text). The fact that no overall enhancement is observed, therefore, may be due to small differences in experimental conditions for the room temperature and 80 K runs, such as the positioning of the pump spot on the bevel. (For room temperature runs, the pump was positioned by maximizing the power of the Fresnel reflection from the bevel, but this was not possible at 80K because the reflection was obscured by the cryostat.)

|  | **Table S1. Enhancement values and buildup center frequencies at 80K** | | | | | | |
| --- | --- | --- | --- | --- | --- | --- | --- |
| Sample | $d$ (μm) | $\theta_{bev}$ (deg) | $f_{0}^{theo}$ (THz) | $f_{0}^{meas}$ (THz) | $L_{\max}$ (mm) | $\bar{\eta}$ | $\eta_{0}$ |
| I | 50 | 59 | 0.56 | 0.58 | 1.4 | 109.4 | 13.4 |
| II | 50 | 53 | 0.87 | 0.65 | 2.4 | 35.3 | 6.4 |
| III | 100 | 59 | 0.28 | 0.26 | 2.5 | 55.3 | 31.3 |
| IV | 100 | 53 | 0.44 | 0.42 | 2.5 | 44.4 | 12.4 |
| V | 100 | 50 | 0.68 | 0.47 | 2.5 | 20.9 | 6.0 |

**
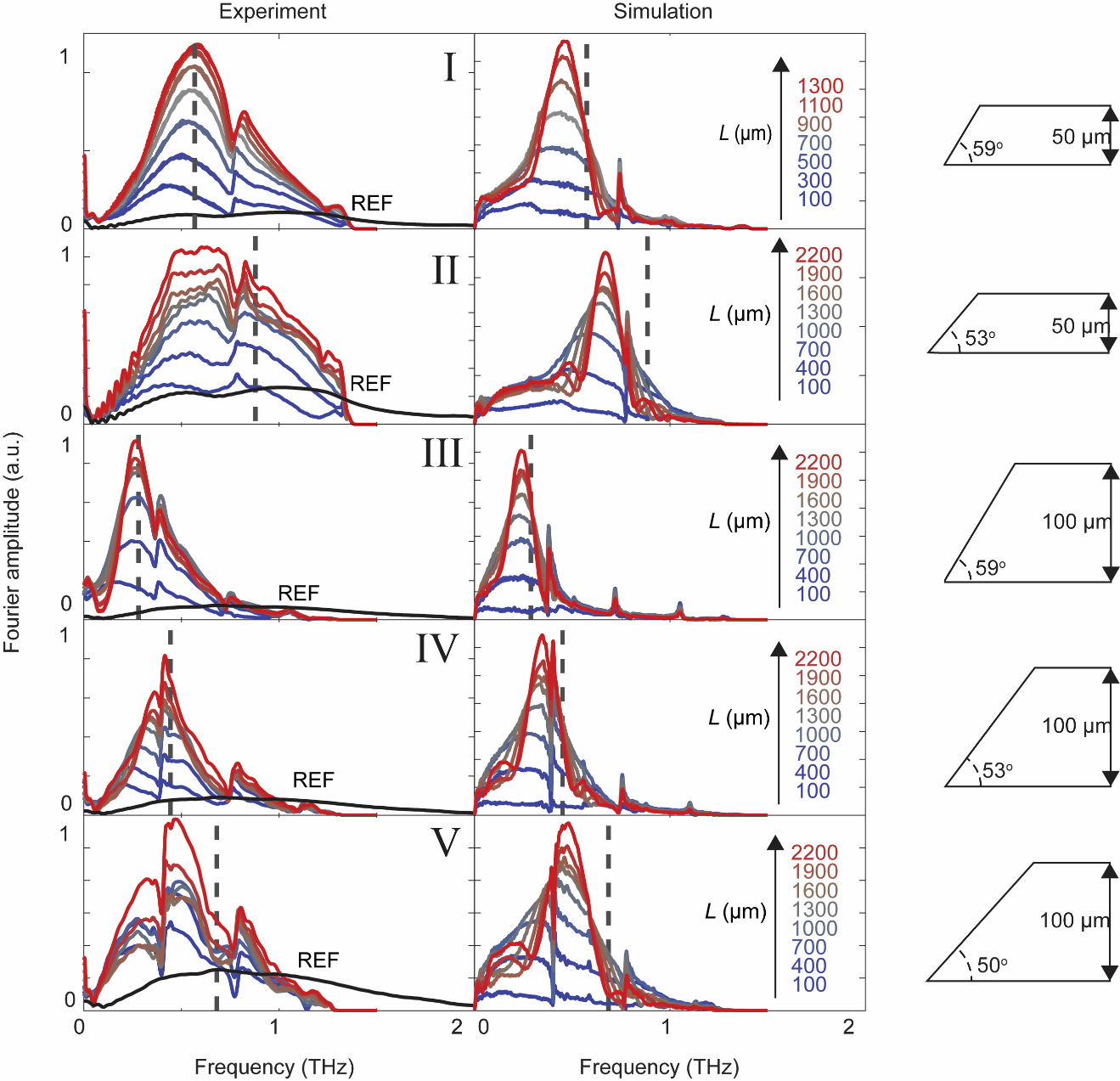
**

**Figure S3.** Buildup of spectral amplitude from both experiment and simulation at 80 K. The corresponding sample for each row of plots is shown on the right. The theoretical buildup frequency is marked by the vertical grey dotted line in each plot. The legend for each plot gives the selected buildup distances, $L$, from $L=100$ μm to ${L=L}_{\max}$. Note that the sharp spectral modulations shown are not present in the main forward-propagating THz pulse that is built up as it propagates. The modulations result from the sequence of backward-propagating THz pulses that reflect off the bevel and then propagate in the forward direction, trailing the main pulse. The narrowband spectral features associated with the sequence of weak pulses overlap with the broad spectrum of the single built-up pulse although the built-up pulse and the pulse sequence do not overlap temporally. See Section S8 for more details.

**
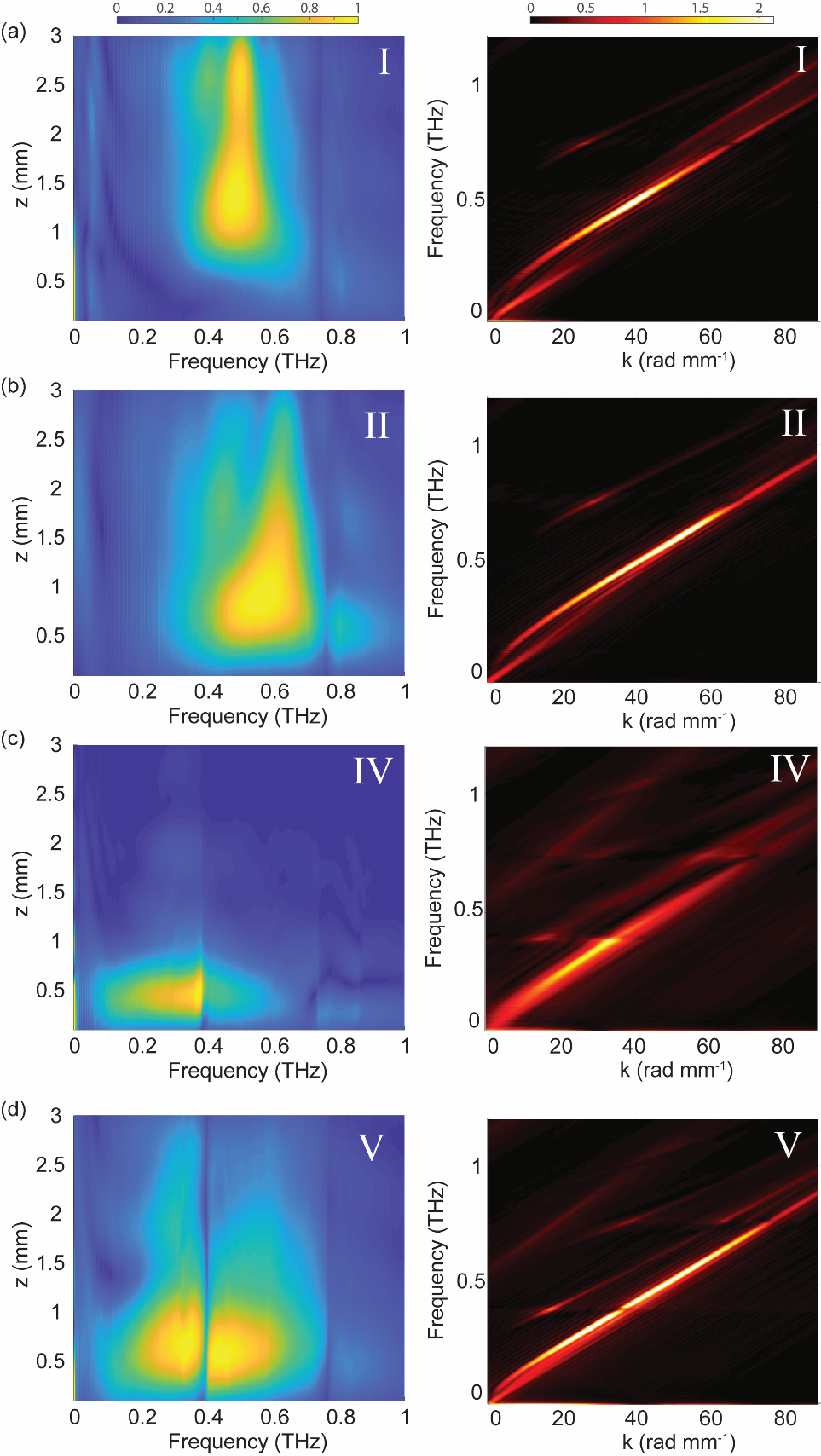
S.4 *Room Temperature Buildup Surfaces and Dispersion Plots***

**Figure S4.** THz buildup surfaces and dispersion plots at room temperature shown for samples I, II, IV, and V. The buildup surface and dispersion plot for sample III are shown in Fig. 2b,c of the main text.

**S.5 *Simulation***

Simulations of the buildup plots (Fig. 3 in main text) were performed using the finite-difference time domain (FDTD) method implemented in MATLAB. The simulation domain was a 0.4 x 3 mm^2^ cross section of the LN slab and air cladding (the third dimension was taken to be of infinite extent due to approximate translational symmetry along the *c*-axis). Perfectly matched layers (PMLs) of 100-μm width (32 grid points) were used at the four boundaries of the simulation to absorb any outgoing waves. LN material dispersion was included in the simulation by including a polarization field^4^, with material parameters taken from Reference 4. The optical pump was modeled as a moving point source whose position was calculated using the bounce angle, LN slab boundaries, and the refractive index of LN at the pump wavelength (800 nm). In the simulation, a new point source appears at each time step displaced by the distance traveled by the pump in that time step. The field radiated by each point source was modeled as a Gaussian pulse with center frequency and bandwidth chosen to match the measured THz reference pulse. The amplitude of the radiated field at each step was taken from a simulation of the nonlinear coupled wave equations in which optical rectification, 3-photon absorption, self-phase modulation, beam divergence, and linear absorption were all taken into account (see Fig. 5 in the main text). This simulation was adapted from the method developed by Ravi et al. for modeling pump depletion effects in TPF THz generation^5^. Briefly, the coupled wave equations for the optical and THz fields are solved in stepwise fashion at discrete points along the THz propagation direction using the Runge-Kutta method. In our case, the slab geometry enters into the simulation through the phase-matching condition, where only the projection of the optical wavevector in the THz propagation is considered,

$$k_{\mathrm{opt}}^{z}\left( \omega\right)=\frac{n\omega}{c}*\frac{1}{\sin\left( \theta_{\mathrm{bnc}} \right)}$$

thus the phase matching relation becomes,

$$k_{\mathrm{opt}}^{z}\left( \omega+\Omega\right)-k_{\mathrm{opt}}^{z}\left( \omega\right)-k_{\mathrm{THz}}\left( \Omega\right)=0$$

which can be written as,

$$n_{\mathrm{opt}}\left( \omega\right)=n_{\mathrm{THz}}\left( \Omega\right)*\sin\left( \theta_{\mathrm{bnc}} \right)$$

where $n_{\mathrm{THz}}\left( \Omega\right)$ is the THz refractive index at frequency $\Omega$. Here $n_{\mathrm{THz}}\left( \omega\right)$ is modified from the bulk to match dispersion in the waveguide geometry.

**S.6 *Geometric Explanation of Modulation Frequencies***

The pump generates THz signals in both the forward propagating and backward propagating directions. In the forward propagating direction we have THz buildup. In the backward propagating direction, a zig-zag pattern of THz signal is generated and propagates towards the bevel. Once the backward traveling wave reaches the bevel a portion is reflected back into the crystal which travels in the same direction as the main pulse. Successive reflection from upward and downward slanting portions of the backward traveling wave pattern give rise to a pulse train, which can be seen in Fig. S5. The pulse train can be represented by a Fourier series with frequencies given by,

$$f_{n}=\frac{n}{T}$$

where $T$ is the temporal spacing between successive reflections (labeled R1, R2, and R3 in Fig. S5) and $n$ is an integer. The frequencies of the first and second-order terms of the Fourier series found in this way are $f_{1}=0.38$ THz and $f_{2} = 0.76$ THz, compared to the observed dips frequencies at $f_{1} = 0.38$ THz and $f_{2} = 0.72$ THz (for a sample with 100 µm thickness). For samples with $d=50$ μm the period, $T$, is halved giving first and second order terms $f_{1}=0.76$ THz and $f_{2}=1.52$ THz which match well the observed frequency dips for those samples.


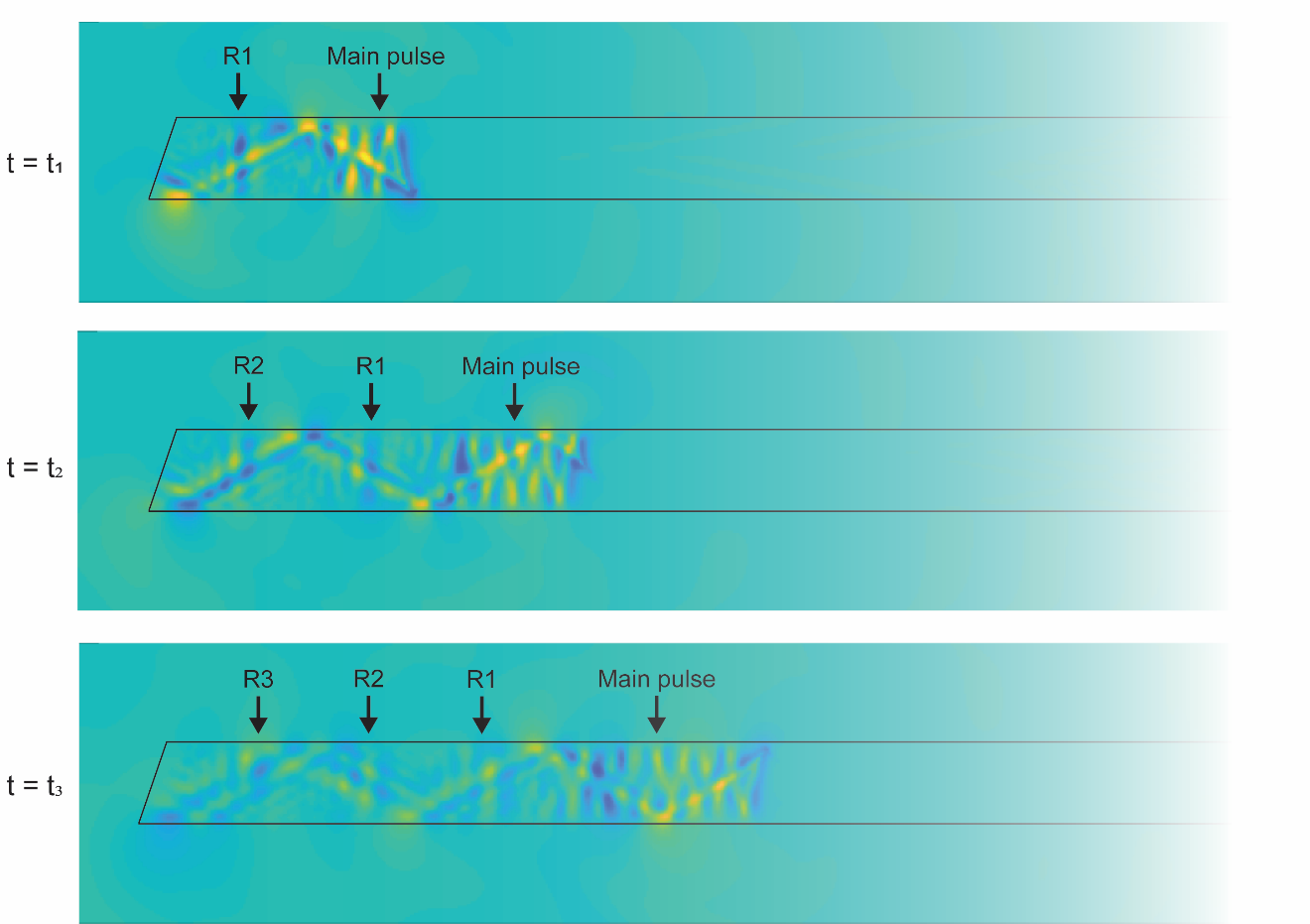


**Figure S5.** Pulse train formed from reflections of backward traveling THz waves from the beveled edge of the LN slab shows at three selected time points. Reflections from successive upward slanting portions of the backward traveling wavefront are denoted as R1, R2, R3, which can be seen lagging behind the main pulse.

**S.7 *Estimation of Overall THz Generation Efficiency***

The overall THz energy was estimated at each distance, $L$, by assuming that the THz wave does not diverge significantly along the c-axis over the course of the buildup and that the profile of the wave in this direction is proportional to the intensity profile of the optical pump. The energy is then given by expression^6^,

$$U_{\mathrm{THz}}\left( L \right)=\frac{1}{2} \int_{-\infty}^{\infty} \partial x \int_{-\infty}^{\infty} \partial y\int_{-\infty}^{\infty} \partial\omega\left[ \epsilon_{0}{c n}_{\mathrm{THz}}\left( \omega\right)^{2}|E{\left( x,y,\omega,L \right)|}^{2} \right]$$

$$E\left( x,y,\omega, L \right)=E_{\mathrm{EO}}\left( \omega,L \right)E_{\mathrm{pump}}\left( x \right)E_{wg}(\omega,y)$$

where $\epsilon_{0}$ is the vacuum permittivity, $E_{\mathrm{EO}}\left( \omega,L \right)$ is the Fourier transform of the EO-sampling trace at location $L$, $E_{\mathrm{pump}}\left( x \right)$ is proportional to $I_{\mathrm{pump}}(x)$ (i.e. the intensity profile of the optical pump in the *x*-direction), and $E_{\mathrm{wg}}\left( \omega,y \right)$ is the TE_0_ dielectric waveguide mode profile at frequency $\omega$. The coordinates used here are defined in Fig 1b. Generation efficiency is obtained by dividing the THz pulse energy, $U_{\mathrm{THz}}(L)$, by the optical pump pulse energy (200 µJ).


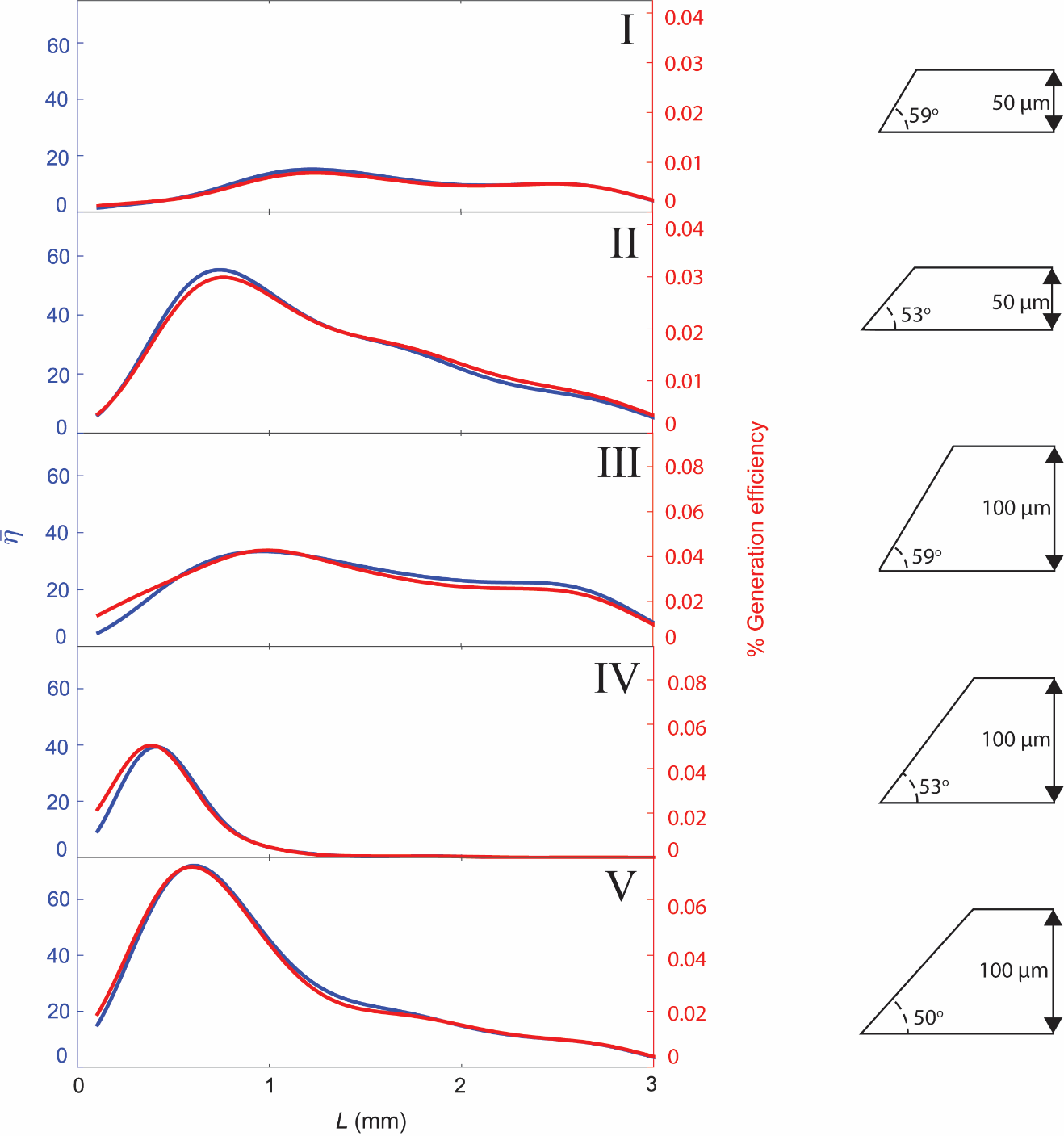


**Figure S6.** Estimated generation efficiency as a function of buildup distance. Comparison with the integrated enhancement factor, $\bar{\eta}$, shows that these quantities can be reasonably treated as proportional to each other. Sample parameters for each of the plots is given by the corresponding diagram on the right.

**S.8 *Time-windowed Buildup Plots***

The spectral modulation observed in the buildup plots shown in Fig. 3 (room temperature) and Fig. S3 (low temperature) is due to reflections of backward-propagating THz signals from the sample bevel as described above. However, the spectral dips that result from Fourier transformation over the entire temporal measurement range should not be interpreted as an absence of these frequencies in the main THz pulse. Because the main pulse arrives at the probe location before the leading edge of the reflected pulse train, the bandwidth of the main pulse can be determined by temporal windowing of the time trace to exclude the pulse train and performing Fourier transformation of only the windowed portion of the time trace. In addition to windowing the experimental data, we performed FDTD simulations as described above, except with an LN slab of infinite extent (no beveled edge), so that backward-propagating waves do not reflect back in the direction of the main pulse. Buildup traces obtained for the windowed experimental data and the infinite slab simulations are shown in Fig. S8. These plots show similar buildup characteristics to those seen in Fig. 3, but with no spectral modulation. The implication for potential spectroscopic applications is that resonances that overlap with the spectral modulation frequencies could still be detected with proper reference subtraction since these frequencies are still contained in the main pulse, and could therefore still be absorbed by a sample. Nonlinear sample responses would often be dominated by the main THz pulse because the subsequent THz signals that yield the narrowband features have relatively small field amplitudes.


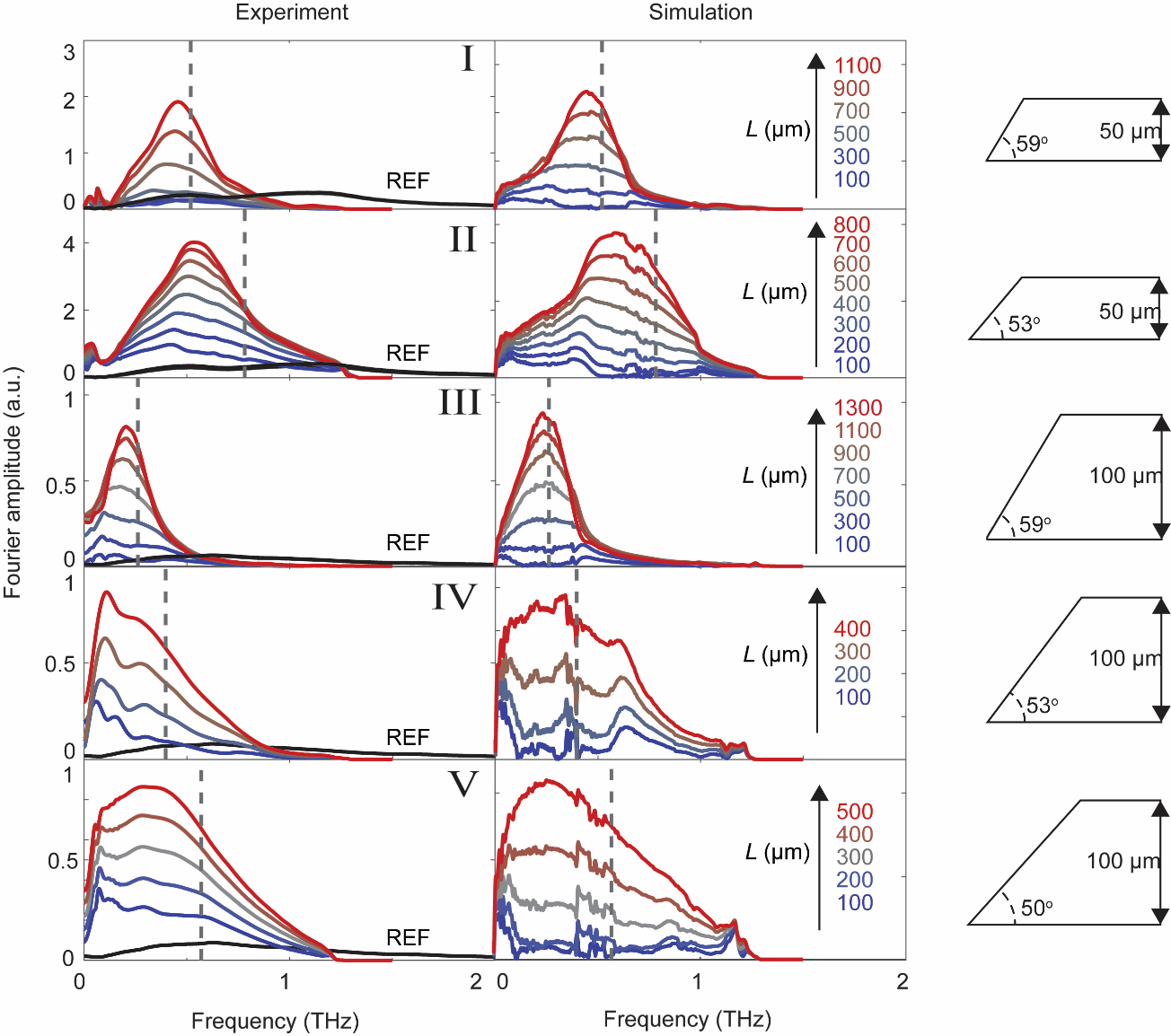


**Figure S7.** Buildup plots for time-windowed experimental traces. The corresponding simulations were performed on slabs of infinite extent (with no bevel) so that no reflection occurs at the bevel.

**References**

1. Yariv, A. & Yeh, P. *Photonics: optical electronics in modern communications*. (Oxford university press, 2007).

2. Wu, X., Zhou, C., Huang, W. R., Ahr, F. & Kärtner, F. X. Temperature dependent refractive index and absorption coefficient of congruent lithium niobate crystals in the terahertz range. *Opt. Express* **23**, 29729–29737 (2015).

3. Huang, W. R. *et al.* Highly efficient terahertz pulse generation by optical rectification in stoichiometric and cryo-cooled congruent lithium niobate. *J. Mod. Opt.* **62**, 1486–1493 (2015).

4. Ward, D. W. Polaritonics: An intermediate regime between electronics and photonics. (2005).

5. Ravi, K., Huang, W. R., Carbajo, S., Wu, X. & Kärtner, F. Limitations to THz generation by optical rectification using tilted pulse fronts. *Opt. Express* **22**, 20239–20251 (2014).

6. Joannopoulos, J. D., Johnson, S. G., Winn, J. N. & Meade, R. D. Molding the flow of light. *Princeton Univ. Press, Princeton, NJ [ua]* (2008).
